# RGD targeting of human ferritin iron oxide nanoparticles can enhance in vivo carotid MRI of experimental atherosclerosis

**DOI:** 10.1186/1532-429X-13-S1-P373

**Published:** 2011-02-02

**Authors:** Toshiro Kitagawa, Hisanori Kosuge, Masaki Uchida, Trevor Douglas, Michael V McConnell

**Affiliations:** 1 Stanford University School of Medicine, Stanford, CA, USA; 2 Montana State University, Bozeman, MT, USA

## Background

Human ferritin (HFn) is a promising nanoscale protein cage platform for molecular/cellular imaging, and we have developed engineered HFn nanoparticles as MRI agents. Inflammation and angiogenesis contribute to atherosclerosis, and RGD is a well-studied ligand of the α_v_β_3_ integrin expressed by activated macrophages and angiogenic endothelial cells.

## Purpose

To evaluate RGD-conjugated HFn-iron oxide nanoparticles for enhanced *in vivo* MRI detection of murine carotid atherosclerosis.

## Methods

### 1) Mice

Fourteen FVB mice underwent left carotid ligation after 4 weeks of high-fat diet and diabetes induction by streptozotocin.

### 2) RGD-conjugated HFn-iron oxide nanoparticles

Using the recombinant human heavy-chain ferritin protein cage, HFn was genetically engineered to display 24 copies of an RGD-4C peptide (CDCRGDCFC) on the exterior surface of the protein cage. Magnetite (Fe_3_O_4_) was encapsulated in interior cavities of RGD-conjugated HFn (RGD^+^ HFn) and non-targeted HFn (RGD^-^ HFn) at loading factors of 5000Fe per cage, giving R2 values of 93 mM^-1^s^-1^ (magnetite diameter: 5-7nm, exterior diameter: 12nm). The injected dose was adjusted to 25mgFe/kg.

### 3) MRI

Two weeks post ligation, mice were imaged on a whole-body 3T MRI scanner (Signa HDx, GE Healthcare) with a phased array mouse coil (RAPID MR International), using a gradient echo sequence (TR/TE=100ms/10ms, slice thickness=1.0mm, FOV=3cm, matrix=256x256, FA=60, NEX=10). Mice were then injected with either RGD^+^ (n=7) or RGD^-^ (n=7) HFn nanoparticles, followed by MRI at 24 and 48 hours post injection. The nanoparticle accumulation was assessed by measuring the extent of T2*-induced reduction in carotid lumen size (% reduction of carotid lumen area).

### 4) Histology

Perl’s iron staining was performed to identify accumulation of the nanoparticles in the carotid lesions.

## Results

**Figure 1:**
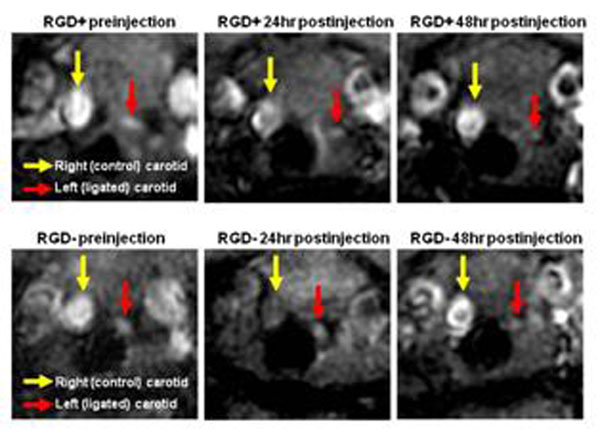


**Figure 2:**
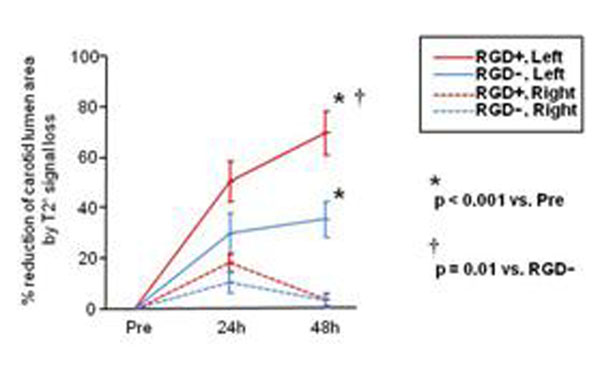


**Figure 3:**
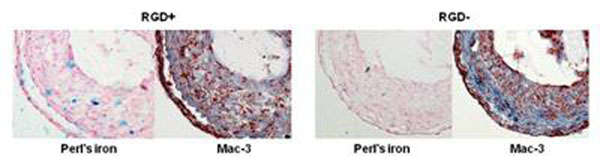


Both RGD^+^ and RGD^-^ HFn nanoparticles caused a reduction in lumen size of the ligated left carotid arteries at 48 hrs due to T2* signal loss (p<0.001 vs. preinjection, Figures 1, 2), but the effect was significantly greater with RGD^+^ HFn (p=0.01 vs. RGD^-^ HFn). There was no significant lumen reduction in the non-ligated (control) right carotid arteries. Perl’s iron staining confirmed greater accumulation of RGD^+^ HFn in the lesion compared to RGD^-^ HFn, primarily in neointimal macrophages (Figure 3).

## Conclusions

Human ferritin protein cage is a versatile nanoparticle imaging platform for *in vivo* cellular/molecular MRI, with enhanced atherosclerosis imaging through multivalent RGD targeting.

